# Clinical Guidelines for the Care of Childhood Cancer Survivors

**DOI:** 10.3390/children1020227

**Published:** 2014-09-12

**Authors:** Emily S. Tonorezos, Tara O. Henderson

**Affiliations:** 1 Departments of Medicine, Memorial Sloan Kettering and Weill Cornell Medical College, 300 East 66th Street, New York, NY 10065, USA; E-Mail: tonoreze@mskcc.org; 2 University of Chicago Medicine Comer Children’s Hospital, 5841 S. Maryland Avenue, MC 4060, Chicago, IL 60637, USA

**Keywords:** survivorship, childhood cancer, guidelines

## Abstract

The Long-Term Follow-Up Guidelines for survivors of childhood, adolescent, and young adult cancers are evidence- and consensus-based guidelines that have been developed and published by the Children’s Oncology Group (COG) Late Effects Committee, Nursing Discipline, and the Patient Advocacy Committee. Originally published in 2004, the guidelines are currently in version 3.0. While the COG guidelines have been praised as a model for providing risk-based survivorship care, adherence has not been uniform. Reasons for this gap include unawareness on the part of the survivor and/or care team as well as disagreement about the individual recommendations. In some cases, the burden of testing (such as annual echocardiography or repeat pulmonary function testing) may be too great. A small number of intervention studies have documented improved adherence to guideline recommendations with dissemination of informational material. Future studies should focus on individualizing screening recommendations, as well as identifying unnecessary testing.

## 1. Introduction

**Figure 1:**
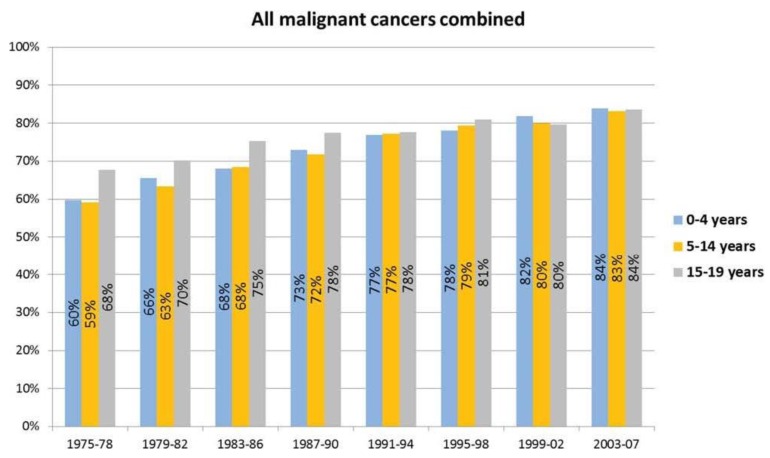
Five-year relative survival is illustrated for children and adolescents who were ages birth to 4 years, 5 to 14 years, and 15 to 19 years at the time of cancer diagnosis. Data is from the Surveillance, Epidemiology, and End Results (SEER) registries with follow-up through 2010. Reprinted with permission from Smith M.A., *et al.* [2].

As a result of the tremendous advances in diagnosis, treatment, and supportive care for childhood and young adult cancer, the survivor population has grown dramatically [1]. While cancer remains the leading disease cause of death among children, for many childhood and young adult cancer patients, the possibility of cure exceeds 80% (Figure 1) [2]. For example, acute lymphoblastic leukemia (ALL), the most common childhood cancer, was uniformly fatal as little as a generation ago [3,4]. Today, with appropriate therapy, five-year survival for children with ALL can be expected to be 90% or greater [1,2]. Contemporary treatment for childhood and young adult cancers includes surgery, chemotherapy, immune therapy, biologic therapy, and/or radiation. Although regimens are optimized to reduce immediate and late effects, long-term survivors of childhood and young adult cancer are at risk for treatment-related late effects throughout the lifespan [5,6,7,8].

**Figure 2:**
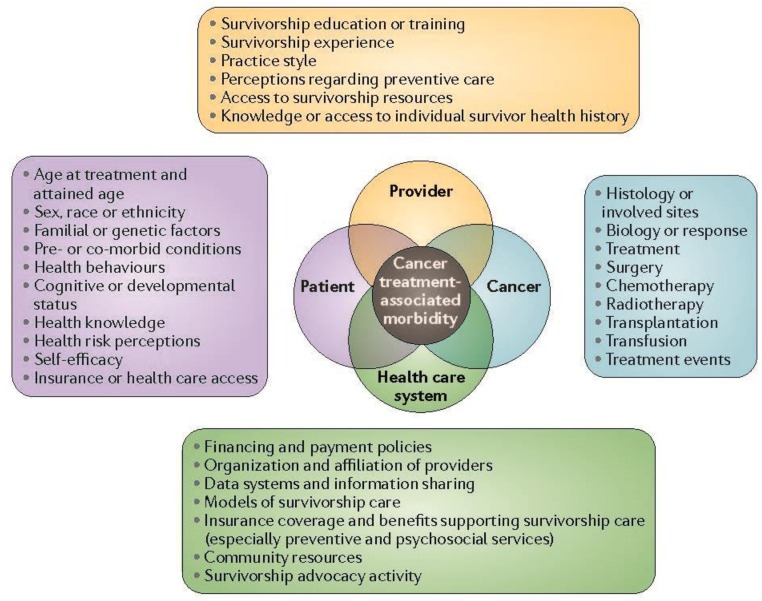
Range of health-related and quality-of-life outcomes among long-term survivors of childhood and adolescent cancers. This figure shows some of the issues that are faced by survivors of childhood and adolescent cancers. GI, gastrointestinal. Reprinted with permission from Robison L.L. and Hudson M.M. [14].

Among long-term adult survivors of childhood cancer, the cumulative incidence of chronic health conditions by 25 years from diagnosis was found to be 66.8% [5]. The most common late effects observed among adult survivors of childhood and young adult cancer are subsequent malignant neoplasms, cardiovascular disease (including coronary artery disease, congestive heart failure, and valvular dysfunction), endocrinopathies, such as abnormal gonadal function, obesity and the metabolic syndrome, and neurocognitive deficits [5,6,7,9,10]. For many late effects, therapy-related risk-factors have been well-characterized. For example, treatment with anthracycline chemotherapy incurs immediate and long-term risks of cardiomyopathy [11,12,13]. Nonetheless, individual variability in susceptibility to treatment toxicities, which may be attributable to age at treatment, attained age, gender, or underlying genetic vulnerability, has also been observed (Figure 2) [14,15,16,17].

Given the expected health risks, survivors of childhood and young adult cancer require comprehensive risk-based health care: close medical attention that is directly coupled to the personal history of cancer and cancer treatment [18]. This need was recognized by the Institute of Medicine (IOM), which, in a 2003 report, called for lifelong-risk-based health care for childhood cancer survivors and for the development of surveillance guidelines [19]. In response to the IOM, *The Long-Term Follow-Up Guidelines for Survivors of Childhood, Adolescent, and Young Adult Cancers* were developed and published by the Children’s Oncology Group (COG) Late Effects Committee, Nursing Discipline, and the Patient Advocacy Committee [20,21]. These clinical practice guidelines draw on evidence-based associations between exposures and late effects to identify high-risk groups that require specialized screening or surveillance. Specific recommendations are made according to exposures. For example, individual recommendations for cardiac surveillance are based on exposure to anthracycline chemotherapy and chest radiotherapy. The COG guidelines are publicly available [20] and are accompanied by a set of patient education materials, called “Health Links”. The guidelines are intended for use starting two or more years after cancer treatment has finished. Similar initiatives have been published internationally [22,23,24,25]. The overall purpose of the guidelines is to raise awareness of potential late effects and to guide clinicians who are caring for survivors of childhood and young adult cancer [20].

In spite of the recognized importance of the COG guidelines, adherence to their recommendations has not been uniform [26,27,28,29,30,31]. In this paper, we will explore awareness and implications of the COG guidelines, as well as the causes for incomplete adherence, including knowledge gaps, economic and social challenges, and test-related anxiety. We will review a small number of studies that have attempted to increase adherence to guideline recommendations, and conclude with suggestions for future research.

## 2. Results and Discussion

### 2.1. Barriers to Adherence

**Figure 3:**
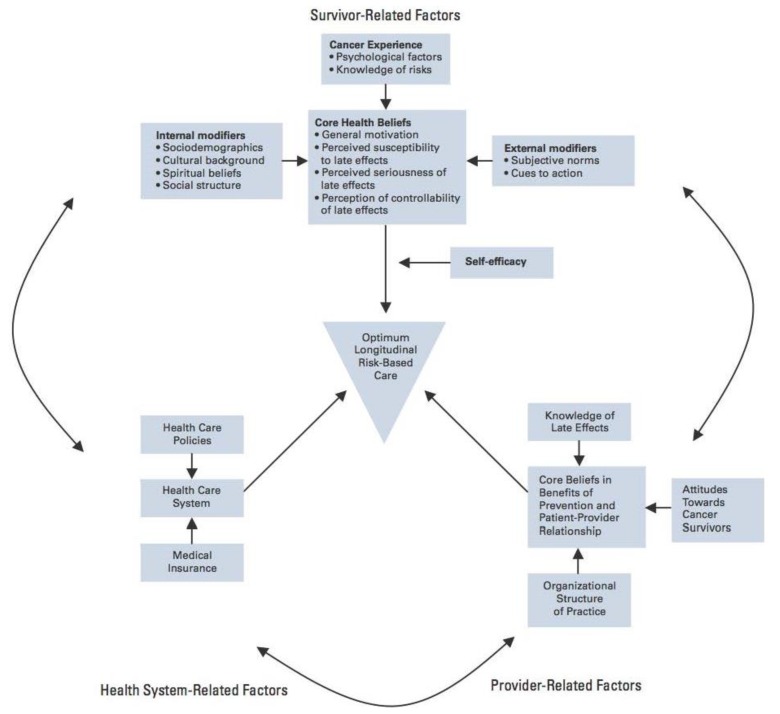
Theoretical model of potential barriers and enablers to the longitudinal cancer-related health care of adult survivors of childhood cancer. Reprinted with permission from Oeffinger, K.C. [33].

In 2006, a second IOM report on childhood cancer survivors noted that this population is “lost in transition” between oncology and primary care-based care; most childhood cancer survivors are not receiving recommended risk-based health care or surveillance [18]. There are many complex and interrelated barriers that contribute to survivors not receiving appropriate follow-up, including survivor, provider, and health care system related barriers (Figure 3) [32,33].

#### 2.1.1. Survivor-Related Barriers

The great majority of adult childhood cancer survivors in the US and Canada are no longer followed by their treating institutions. Over 85% of adult survivors report receiving their health care in the community with a primary care provider and a significant proportion of them are not receiving appropriate risk-based screening [29,30]. For example, Nathan and colleagues examined childhood cancer survivors at high risk for breast cancer, colorectal cancer, and skin cancer due to radiation exposure and found most were being screened far less than recommended by the guidelines; less than 50%, 15% and 30% of these survivors were receiving recommended mammography, colonoscopy and skin exams, respectively [26]. One factor contributing to survivors not being engaged in appropriate healthcare is that many survivors are not well informed regarding their prior cancer, its treatment and the potential risks for sequelae. Survivors may not be able to accurately recall their treatment history, may not have access to their medical records, and may not be aware of the risks of late effects. In one investigation from of the North American Childhood Cancer Survivor Study, only 35% of the survivors recognized that serious health problems are associated with cancer treatment [34].

Psychological barriers may contribute to survivors not transitioning to appropriate long-term follow up care and engaging in risk-based surveillance as adults. Survivors may be over-dependent on their families, may have anxiety, or may lack in caregiver trust. While studies suggest that many survivors are coping well, some struggle to cope with life after cancer [35,36,37,38]. Indeed, some survivors have post-traumatic stress disorder, with symptoms inducing re-experiencing, arousal and avoidance behaviors [39,40,41]. This avoidance can interfere with obtaining appropriate health care and information, compounding the issue of knowledge gaps in the population. Anxiety may also negatively impact adherence to cancer screening in high risk populations such as women at high risk for breast cancer following exposure to chest radiation, as has been demonstrated among women at high risk for breast cancer due to familial risk [42]. Likewise, some survivors have cognitive or developmental delays which further impact their knowledge of their past care, their ability to access and engage in appropriate long-term care in the adult setting, and how they communicate their past cancer and its treatment with their adult providers [43,44,45,46,47].

Recognizing that survivors need information regarding their past cancer for their current and future health care, the 2006 IOM report recommended that all cancer survivors and their primary care providers be provided a survivorship care plan, which includes a treatment summary, contact information for the treating oncologist, and guidelines for follow up care, including recommendations for risk-based surveillance for late effects and second cancers [18]. However, only 15% of adult childhood cancer survivors report having received the treatment summary element of a survivorship care plan [29].

#### 2.1.2. Provider-Related Barriers

Several large studies have evaluated the knowledge and preferences of specialty and primary care physicians regarding care of childhood cancer survivors and the COG guidelines [48,49,50]. In 2013, Nathan *et al.* [48] published the results of a survey of over one thousand family physicians in the United States and Canada. In general, respondents cared for very few childhood cancer survivors; the average was ≤2 childhood cancer survivors in the past five years. Comfort in caring for this population and correct identification of appropriate, risk-based screening were uncommon. Only nine of the 1092 respondents (1%) preferred to care for survivors independently. Importantly, family physicians were unfamiliar with the clinical guidelines, and reported that access to clinical guidelines would be useful [48]. More recently, Suh *et al.* reported results of a survey of 2000 general internists in the United States [49]. While approximately half of the responding internists reported caring for a childhood cancer survivor, they felt, on average, “somewhat uncomfortable” with caring for survivors and were, on average “somewhat unfamiliar” with the available clinical guidelines [49]. The results of both of these studies suggest that lack of knowledge of the COG guidelines among primary care physicians is a barrier to their effectiveness.

Similar knowledge gaps have been described among pediatric oncologists. In a survey of pediatric oncologists in the North American consortium, the Children’s Oncology Group, only 33% of respondents correctly answered vignette-based questions about the surveillance recommendations for breast cancer, cardiomyopathy and thyroid function [50]. These knowledge gaps among pediatric oncologists may result in lack of referral for COG guideline-driven risk-based care.

In general, guidelines may have a limited effect on changing physician behavior. Cabana and colleagues suggest through a systematic review of the literature that barriers to physicians adhering to guidelines include not just lack of awareness or lack of familiarity, but also disagreement, poor self-efficacy (doubt in the ability to achieve a goal), low outcome expectancy, inability to overcome the inertia of previous practice, and external barriers [51]. Nonetheless, physician recommendation is vital to achieving appropriate screening. In a study of breast cancer surveillance practices among 551 exposed to chest radiation in the Childhood Cancer Survivor Study, 63.5% women aged 25 to 39 and 23.5% of women age 40 to 50 years had not had mammogram screening in the past two years [31]. Screening rates were highest among survivors who reported a physician recommendation, highlighting that appropriate risk-based surveillance depends on provider awareness of second cancer risks and surveillance recommendations. Yet, less than 20% of general internists and family physicians who report having seen childhood cancer survivors in their practice have received a survivorship care plan or treatment summary to guide screening recommendations [48,49].

#### 2.1.3. Health System-Related Barriers

To date, a barrier for many young adult survivors of childhood cancer in the US to receiving long-term follow up care has been insurability [52]. Until the enactment of the Affordable Health Care Act (ACA), employers largely provided health benefits for young adults as survivors aged out of parental or public insurance plans, which could be costly or inadequate [53]. With the ACA, young adults may stay on plans longer and can obtain insurance through the exchanges [54]. Pre-existing conditions no longer prevent survivors from obtaining appropriate insurance. However, given the high cost of many of the tests recommended for risk-based surveillance, many of the tests may not be covered or only partially covered by insurance policies, and knowledge of insurance coverage among childhood cancer survivors is incomplete [55].

For example, given significantly elevated rates of breast cancer at an early age, both the Children’s Oncology Group and the American Cancer Society recommend that women who were exposed to chest radiotherapy for their childhood cancer initiate breast cancer surveillance with breast MRI and mammography at an early age [56,57]. Some insurance policies often will not provide reimbursement for the cost of these tests or will cover the test after a high deductable has been reached, incurring a high financial burden on survivors who comply with these recommendations [58]. Lastly, even with insurance coverage, some policies often have limited numbers of covered physicians, and finding a physician with appropriate willingness and expertise to follow this high-risk population may be difficult [59,60]. As has been the case prior to the ACA, access and coverage may disproportionately affected racial and ethnic minorities and those in lower socio-economic strata.

### 2.2. Improving Adherence

#### 2.2.1. Refinement of the Guidelines

While awareness of the COG guidelines requires attention, refining the guidelines may also improve effectiveness. Several recent studies have worked towards enhancing the guidelines via investigations of yield and cost effectiveness [6,61]. Landier and colleagues reported on the yield of the COG guidelines for 370 childhood cancer survivors who underwent nearly 5000 screening tests during the course of 1188 clinic visits. While some tests, such as thyroid function testing, audiometry, DXA scanning, serum ferritin, and pulmonary function testing resulted in clinical relevant findings, many tests, including screening CBC (for therapy-related leukemia) and EKG were of low yield [61]. Recommendations for frequent echocardiography, as currently suggested by the COG guidelines for many survivors, may also benefit from refinement. Analyses using mathematical modeling by Yeh, Nohria, and Diller [62], as well as Wong and colleagues [63] have suggested that performing echocardiographic screening less often than currently recommended by the COG guidelines may be nearly as effective in reducing CHF risk.

#### 2.2.2. Interventions to Improve Adherence

In order to minimize morbidity and mortality in childhood cancer survivors, it is imperative to develop intervention studies aimed at improving compliance with recommendations for at-risk survivors. When developing these interventions it is important to consider the barriers to adherence as outlined above, including provider, survivor and health system related factors. A survivorship care plan could ameliorate many of these barriers. Oeffinger and colleagues examined the impact of a mailed one-page survivorship care plan among 72 Hodgkin lymphoma survivors in the CCSS on communicating risk and improving rates of risk-based surveillance. At six-months, 41% of survivors reported having a recommended mammogram and 20% reported having a recommended echocardiogram [64].

Several randomized intervention trials aimed at improving compliance with surveillance guidelines in high-risk survivors are ongoing. These NCI-funded studies are each being conducted within the Childhood Cancer Survivors Study and include: (1) improving breast cancer surveillance rates in women exposed to chest radiation for a childhood cancer (EMPOWER: Oeffinger, Principal Investigator); (2) improving cardiovascular screening among childhood cancer survivors treated with cardiotoxic therapy and at high risk for left ventricular dysfunction (ECHOS: Hudson and Cox, Co-Principal Investigators); and (3) improving skin protection and skin cancer detection among survivors treated with radiation and at elevated risk of skin cancer (Geller, Principal Investigator). The EMPOWER study is examining the impact of a motivational interview to improve rates of breast cancer surveillance as compared to standard mailed education materials among 330 women exposed to chest radiotherapy for their childhood cancer. The ECHOS study has randomized 472 childhood cancer survivors at high risk for left-ventricular dysfunction due to exposure to anthracyclines and/or chest directed radiotherapy to examine the impact of motivational interview via telephone in addition to a Survivorship Care Plan as compared to a mailed Survivorship Care Plan alone in completing recommended surveillance echocardiograms. Dr. Hudson presented her preliminary findings at the 2014 American Society of Clinical Oncology Annual Meeting and reported that 52% of those in the experimental arm completed recommended cardiac surveillance as compared to 22.3% in the control arm [65]. Dr. Geller’s study is aimed at examining the impact of teledermatology in combination with patient activation and education on early skin cancer detection among survivors exposed to radiation [18].

## 3. Conclusions

As more childhood cancer patients are cured, and as the current population of survivor ages, the number of survivors who need risk-based long-term follow up care will increase. With this growth in population, the need for informed, risk-based care will increase, and the role of the COG guidelines will also grow. Testing the guidelines, to determine if the recommendations are indeed ameliorating the chronic health conditions and second cancers associated with childhood cancer and its treatment will be necessary. As described previously, informative research by Hudson and colleagues [6], Landier and colleagues [61], Yeh, Nohria and Diller [62], as well as Wong and colleagues [63], have begun to clarify which screening tests will be most valuable and productive. Primary care providers may need to tailor screening approaches to the individual patient. In addition, the findings from these studies will be necessary to consider in future iterations of the guidelines.

Another vital area of focus in childhood cancer survivorship research is refining individual risk for late effects and second cancers to inform long-term surveillance recommendations. For example, Kovalchik and colleagues developed an absolute risk prediction model for second primary thyroid cancer among five-year survivors of childhood cancer, which considers survivors basic treatment information [66]. Similarly, investigators are presently developing risk prediction models for breast cancer in women exposed to chest radiotherapy and for cardiomyopathy in survivors exposed to chest radiotherapy and/or anthracycline chemotherapy, taking into consideration factors such as family history and other exposures.

The Children’s Oncology Group continues to update the long-term follow-up guidelines, with an updated version expected in late 2014. Improving knowledge and familiarity with the COG guidelines among primary care practitioners will be critical for ensuring their impact. Additionally, achieving international consensus on appropriate screening is important; an international endeavor to harmonize potentially disparate guidelines for childhood, adolescent and young adult cancer survivors is currently underway [67]. Finally, future studies to identify barriers to adherence in different practice settings, including those that are resource-poor, and to help eliminate unnecessary testing, are needed.
